# Analysis of Immunophenotypic Changes during Ex Vivo Human Erythropoiesis and Its Application in the Study of Normal and Defective Erythropoiesis

**DOI:** 10.3390/cells12091303

**Published:** 2023-05-02

**Authors:** Shobhita Katiyar, Arunim Shah, Khaliqur Rahman, Naresh Kumar Tripathy, Rajesh Kashyap, Soniya Nityanand, Chandra Prakash Chaturvedi

**Affiliations:** 1 Stem Cell Research Center, Department of Hematology, Sanjay Gandhi Postgraduate Institute of Medical Sciences, Lucknow 226014, India; katiyarshobhita@gmail.com (S.K.); arunimshah@gmail.com (A.S.); 2 Department of Hematology, Sanjay Gandhi Postgraduate Institute of Medical Sciences, Lucknow 226014, India; drkhaliq81@gmail.com (K.R.); nareshtripathy@gmail.com (N.K.T.); rajkashyapmd@yahoo.co.in (R.K.); soniya_nityanand@yahoo.co.in (S.N.)

**Keywords:** erythropoiesis, flowcytometry, marker panels

## Abstract

Erythropoiesis is a highly regulated process and undergoes several genotypic and phenotypic changes during differentiation. The phenotypic changes can be evaluated using a combination of cell surface markers expressed at different cellular stages of erythropoiesis using FACS. However, limited studies are available on the in-depth phenotypic characterization of progenitors from human adult hematopoietic stem and progenitor cells (HSPCs) to red blood cells. Therefore, using a set of designed marker panels, in the current study we have kinetically characterized the hematopoietic, erythroid progenitors, and terminally differentiated erythroblasts ex vivo. Furthermore, the progenitor stages were explored for expression of CD117, CD31, CD41a, CD133, and CD45, along with known key markers CD36, CD71, CD105, and GPA. Additionally, we used these marker panels to study the stage-specific phenotypic changes regulated by the epigenetic regulator; *Nuclear receptor binding SET Domain protein 1* (*NSD1*) during erythropoiesis and to study ineffective erythropoiesis in myelodysplastic syndrome (MDS) and pure red cell aplasia (PRCA) patients. Our immunophenotyping strategy can be used to sort and study erythroid-primed hematopoietic and erythroid precursors at specified time points and to study diseases resulting from erythroid dyspoiesis. Overall, the current study explores the in-depth kinetics of phenotypic changes occurring during human erythropoiesis and applies this strategy to study normal and defective erythropoiesis.

## 1. Introduction

Definitive erythropoiesis is orchestrated by a small pool of hematopoietic stem cells (HSCs) in adult bone marrow. During this process, the HSCs proliferate and differentiate into multipotent stem cells (MPPs), which undergo successive clonal divisions to give rise to committed hematopoietic progenitors: common myeloid progenitors (CMPs), granulocyte/macrophage progenitors (GMPs), and megakaryocyte/erythroid progenitors (MEPs) [1,2]. Unileniage CMPs are thought to give rise to GMPs and MEPs [3], and these bivalent MEPs give rise to early erythroid precursors, burst-forming units erythroid (BFU-E). BFU-E undergo limited self-renewal divisions and differentiate into colony-forming units erythroid (CFU-E) [4]. Terminal erythroid differentiation begins as CFU-E mature into proerythroblasts (Pro-EB), which finally undergo 3–4 mitosis to generate sequentially basophilic (Baso-EB), polychromatic (Poly-EB), and orthochromatic erythroblasts (Ortho-EB). Ortho-EB expel their nuclei to produce reticulocytes, which finally culminate in RBCs in circulation. This whole progression of erythropoiesis gradually results in genotypic and phenotypic changes [5].

With the advent of flow cytometry in the 1980s, a lot of progress has been made in identifying phenotypic changes with the help of cell surface markers for studying erythropoiesis. The phenotypic changes lead to the identification of different stages of erythroid differentiation. These stages can be identified either in bone marrow or by ex vivo studies. The ex vivo culture represents a concise model that mimics changes occurring during erythropoiesis in the bone marrow niche [6,7]. In mice, latent studies have identified the precursor stages, i.e., BFU-E and CFU-E as c-Kit+CD45+Ter119−CD71low and c-Kit+CD45−Ter119−CD71high cells in the fetal liver [8], while in bone marrow CFU-E are characterized as Lin−cKit+Sca-1-IL-7Rα-IL3Rα-CD41−CD71+ cells [9]. Further terminal stages have been identified by using Ter119, CD71, and FSC as markers in in-vivo studies [10]. To better resolve the heterogeneity, Liu et al. replaced CD71 with CD44, as the decrease in CD44 was more progressive with each cell division as compared to the little change in CD71 expression [11]. 

Compared to the extensive work on mouse erythropoiesis, human erythropoiesis is less explored, except for a few studies [12,13,14]. Li et al. characterized human BFU-E and CFU-E as CD45+GPA−IL-3R−CD34+CD36−CD71 low and CD45+GPA−IL-3R−CD34+CD36+CD71 high cells in both cultured cells and in bone marrow [15]. GPA, band 3, and α4 integrin have been used as surface markers to describe the terminal stages both in-vitro and in bone marrow [16]. In addition to FSC, markers CD71, CD36, CD117, and CD105 have also been used to detect the terminal stages in human bone marrow [17]. However, these studies have limitations to decipher the complete erythroid continuum, thus indicating a need for a more robust method that can quantify specific cell types at different stages of erythropoiesis beginning from HSPCs to RBC maturation based on specific immunophenotypic properties. The studies on ex vivo erythroid differentiation have been intrinsic to studying the mechanistic and molecular changes occurring during the erythropoiesis [6,18] however, a detailed characterization and kinetics of the changes in the expression of cell surface markers during erythroid differentiation has not been explored in depth. 

Therefore, to address this unmet need, in the present study, we have designed and used comprehensive immunophenotypic marker panels to study the kinetics of stage-specific transitions from uncommitted (HSCs, MPPs, lymphoid-primed multipotent progenitors (LMPPs)) and committed progenitors (CMPs, GMPs, MEPs) to RBCs using ex vivo culture. We then accessed the lineage directionality of these precursors obtained from HPSCs by examining the median fluorescence intensity (MFI) of CD133, CD45, CD31, CD41a, CD105, CD117, CD71, and CD36 within these cells. Secondly, we studied the dynamics of phenotypic changes occurring from the BFU-E to CFU-E transition using the strategy as described by Li et al. [15]. To further examine the putative occupancy of markers within these cells, we used CD31, CD117, and CD105 to explore this finding. Thirdly, based on changes in the expression of CD117 and CD105 on CD71+ve cells, we identified Pro-EB, Baso-EB, and Poly-Ortho-EB. Poly and Ortho-EB were further segregated based on the expression of GPA and FSC. We further explored the expression of CD36 and GPA in terminally differentiating erythroblasts which currently remains ambiguous. Also, CD31+CD45+ erythro-myloid progenitors have been shown to give rise to RBCs [19], but the expression of CD31 at the terminal stages remains unknown, therefore we explored the expression of CD31 on precursors as well as in terminal stages of differentiation.

Lastly, to evaluate the applicability of marker panels to study altered erythropoiesis, we applied our immunophenotyping strategy to study the stage-specific phenotypic changes regulated by the epigenetic regulator *NSD1* during ex vivo erythropoiesis. Knocking down of *NSD1* at days 2, 4, and 12 (on the progenitor and terminal stage progenitors) of differentiation followed by immunophenotyping revealed the alternation at all stages thus, highlighting *NSD1* is vital for erythroid differentiation [20]. We further used our immunophenotyping strategy in diseases arising from defective erythropoiesis i.e., in MDS (erythroid dysplasia) [21] and PCRA [22]. The patients carrying MDS or PRCA showed erythroid defects at progenitor and terminal stages thus, indicating that our immunophenotypic strategy can be also used to study dysregulated/defective erythropoiesis. 

## 2. Materials and Methods

### 2.1. Purification and Culture of CD34+ Cells

G-CSF-mobilized peripheral blood samples were collected from healthy volunteers aged between 18–30 years after gaining informed ethical consent. The study was conducted according to the guidelines of the Declaration of Helsinki and approved by the Institutional Ethics Committee (IEC) and Institute Committee for Stem Cell Research (IC-SCR) of Sanjay Gandhi Post Graduate Institute of Medical Sciences (SGPGIMS), Lucknow (IEC code: 2021-12-SRF-118 and IC-SCR code: 2021-02-SRF-EXP-3). CD34+ cells were enriched from mobilized peripheral blood samples by magnetic activated cell sorting (MACS) using CD34+ immunomagnetic beads (Miltenyi Biotech, Bergisch Gladbach, Germany, Cat no. 130-046-703). CD34+ enriched cells were analyzed using a BD FACS Lyric^TM^ system, San Jose, CA, USA) for CD34+ expression using Mouse anti-Human CD34 antibodies and Mouse anti-Human CD45 antibodies. The 95–98% pure HSPCs were cultured in serum-free media for 26 days, according to the 4-step protocol as described by Palii et al. (2011) [7] with the following modifications: the concentration of EPO (Prospec, Ness-Ziona, Israel, Cat no. CYT-201) was increased from 3UI/mL to 6UI/mL from day 4 until day 15. From day 15 onwards, cytokine-depleted co-culture was supplemented with 25% heat-inactivated human AB serum (Sigma-Aldrich, Burlington, VT, USA, Cat. No. H3667) for better sustenance of cells. The cells in the culture were counted every second day and were monitored for viability during ex vivo erythroid differentiation.

### 2.2. Benzidine Staining

A total of 1 × 10^6^ cells were incubated for 5 min at room temperature in a benzidine solution containing 0.4% benzidine dihydrochloride (Sigma-Aldrich, Darmstadt, Germany Cat. No. B3383) in 12% glacial acetic acid (Merck EMPLURA^®^, Burlington, VT, USA, Cat. No. 1.93402.0521) and 0.3% H_2_O_2_ (Fisher Scientific, Mumbai, India, Cat. No. 15465) (added before use). Slides were imaged at 20X using an Olympus IX53 inverted microscope (Tokyo, Japan).

### 2.3. Colony-Forming Assays

A total of 1 × 10^4^ CD34+ cells were resuspended in 300 μL of IMDM medium (Sigma-Aldrich, St. Louis, MO, USA, Cat. No. I3390) containing 2% FBS (Gibco^TM^, Brazil, SA, Cat. No. 10437028) and 1% Pen/Strept (Gibco^TM^, New York, NY, USA, Cat. No. 15140-122) Cells were seeded in a 35 mm cell culture dish with 3 mL of MethoCult^TM^ medium (STEMCELL Technologies^TM^, Vancouver, Canada, Cat no. H4434 Classic) containing 1% Pen/Strept (Gibco^TM^, New York, NY, USA, Cat. No. 15140-122) and were pre-warmed in a 37 °C water bath for 2 min. A 35-mm dish containing cells in MethoCult^TM^ was kept in a 100-mm dish containing an uncovered 35-mm culture dish with sterile water to maintain humidity. The dish was incubated in a 5% 37 °C CO_2_ incubator for 12–14 days. BFU-E and CFU-E were counted and imaged using an Olympus IX53 inverted microscope (Tokyo, Japan).

### 2.4. May–Grunwald–Giemsa Staining

A total of 1 × 10^6^ cells were harvested and washed using 1X PBS fixed in 100% methanol for 10 min. Slides were prepared and stained by May–Grunwald (Sigma Aldrich, St. Louis, MO, USA, Cat no. MG-63590) and Giemsa stain (Sigma-Aldrich, St. Louis, MO, USA, Cat no. Giemsa-48900) as per the standard Giemsa protocol provided by the manufacturer. Slides were imaged at 20X magnification using an Olympus IX53 inverted microscope (Tokyo, Japan).

### 2.5. Hoechst and Syto^TM^ 16 Staining

A total of 1 × 10^6^ cells were washed and fixed in 100% methanol for 10 min. Slides were prepared and stained with 5mg/mL Hoechst stain (Thermo Scientific, Rockford, IL, USA, Cat. No 62249) as per the manufacturer’s protocol. Slides were imaged at 40× in a standard DAPI filter set using a Carl Zeiss LSM880 confocal microscope with Airyscan (Peabody, MA, USA). 

For Syto^TM^ 16 staining, 5 × 10^5^ cells were washed with 1X PBS containing 1% BSA and were stained with SYTO^TM^ 16 Green Fluorescent Nucleic Acid Stain (Life Technologies, Oregon, OR, USA, Cat no. S7578) as per manufacturer’s instructions. Stained cells were analyzed on BD FACSLyric^TM^ system (San Jose, CA, USA).

### 2.6. FACS Analysis

A total of 2 × 10^5^ cells were harvested on an alternate day and washed with a FACS buffer containing 1X PBS, 5 mM MgCl_2_, and 1% BSA. Cells were stained with a cocktail of antibodies for each panel as mentioned in Table 1, Table 2 and Table 3 as per the manufacturer’s recommendation in a staining buffer containing 1X PBS, 0.09% BSA, 5 mM MgCl_2_, 50 ug/mL DNAse I, and 5 μL of FCR blocking reagent. They were incubated for 30 min at room temperature. The cells were washed and resuspended in FACS buffer and were analyzed on a BD FACSLyric^TM^ system (San Jose, CA, USA). BD FACSuite^TM^ software (version 1.5) was used for gating and analysis.

### 2.7. Lentivirus Preparation and Cell Transduction

Two small hairpin sh-RNA (one coding for CDS and one for 3′UTR region) against *NSD1* or a scrambled sequence (control) were cloned in the pLKO.1 vector (sequences available in Table 4). Lentiviral particles were produced by co-transfection of 293FT cells (ThermoFisher^TM^, New York, NY, USA, Cat no. R70007) with sh-RNA-expressing vector, pVSVG, and pdR8.9 using Lipofectamine^TM^ 2000 transfection reagent (ThermoFisher^TM^, Carlsbad, CA, USA, Cat no. 11668019). The viral culture supernatant was concentrated as previously described. Cells were transduced with lentivirus in the presence of 1.5 mg/mL LentiBOOST^®^-P (SIRION Biotech, Munich, Germany) and spinoculated at 1200 g for 75 min. Fresh media were added after 12 h of transduction. Cells were cultured as described in (Section 2.1) under “Methods”.

### 2.8. RNA Extraction and qPCR Analysis

Total RNA was isolated using the Quick RNA^TM^ Micro Prep Kit (Zymo Research, Orange, CA, USA, Cat. No. R1050) according to the manufacturer’s instructions. One microgram of RNA was converted to cDNA using the high-capacity cDNA reverse transcription kit (Applied Biosystems, Foster City, CA, USA, Cat. No. 4368814). A real-time quantitative PCR was performed on the BioRad^TM^ CFX 96 real-time instrument (Hercules, CA, USA) using the Hot Fire Pol Eva-green qPCR mix (Solis BioDyne, Tartu, Estonia, Cat no. 08-24-0000S) with gene-specific primers (sequences mentioned in Table 5). *GAPDH* was used for normalization, and relative quantification was undertaken as described previously [23].

### 2.9. Statistical Data Analysis

The data are expressed as mean ± standard deviation (SD) or standard error of mean (SEM). Statistical evaluations between 3 independent experiments were performed using Student’s *t*-test and *p* < 0.05 was considered to indicate statistical significance.

## 3. Results

### 3.1. Establishment of Erythroid Differentiation

**Figure 1:**
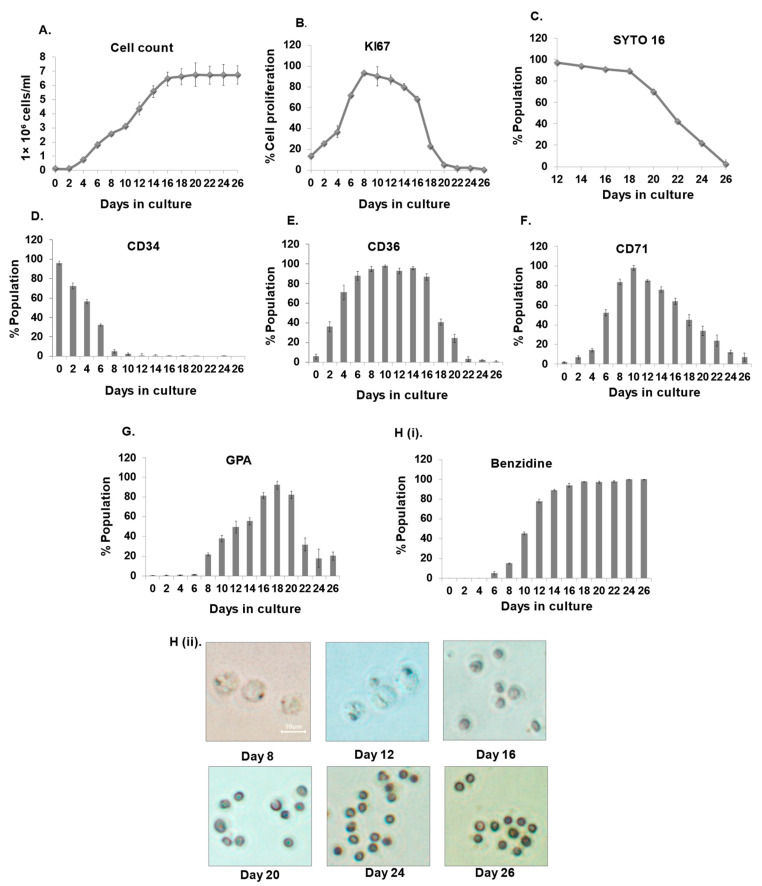
Characterization of ex vivo erythroid differentiation. (**A**,**B**) Analysis of cell amplification and proliferation: cells were counted and stained using KI67 on alternate days to measure amplification and proliferation, respectively. (**C**) Analysis of enucleation in maturing erythroblasts by Syto-16 staining using FACS. (**D**–**G**) Analysis of hematopoietic and erythroid-specific markers, i.e., CD34, CD36, CD71, and GPA, during erythroid differentiation at respective time points by FACS. (**H**(**i**,**ii**)) Analysis of hemoglobinization by benzidine staining on the indicated days. Error bars indicate mean ± SD of (n = 3) independent experiments conducted on HSPC samples obtained from three healthy individuals.

CD34+ HSPCs were differentiated to the erythroid lineage and monitored every other day to measure cell count and proliferation. We observed that the cells began to multiply on day 2 and reach the threshold by day 16 (Figure 1A). Further cell proliferation was observed to be maximal at day eight and declined as erythroid differentiation progressed (Figure 1B). Enucleation in maturing erythroblasts starts on day 18 and reaches 96–98% by day 26, as observed by Syto16 staining (Figure 1C). A FACS analysis of hematopoietic and erythroid markers showed that the hematopoietic marker CD34 tends to decline from day 2 and is completely lost by day 10 of differentiation (Figure 1D). We found that expression of CD71 and CD36 gradually increased in the initial days and later decreased (Figure 1E,F). This finding was similar to what had previously been reported [7]. Several studies state GPA as a late erythroid marker [7,24]; however, we observed that GPA expression is first observed on day 8 and was found to be maximal at day 18 and decreases further (Figure 1G). Moreover, benzidine staining revealed that hemoglobin accumulation appears on day eight and gradually increases during differentiation (Figure 1H(i,ii)). Furthermore, the erythroid-specific genes (*β-globin*, *GATA1, KLF1*) were monitored at the molecular level, which progressively increased during erythroid progression and subsequently decreased as the cells experienced maturation and enucleation (Figure S1).

### 3.2. Kinetics of Committed and Uncommitted Progenitors during Erythroid Differentiation

**Figure 2:**
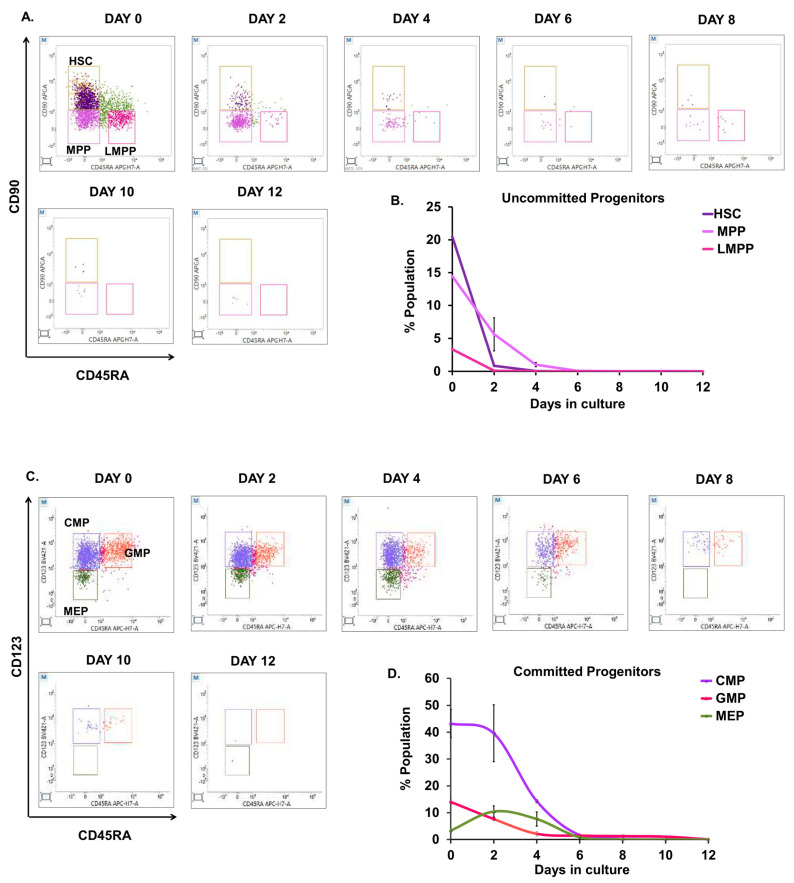
Characterization of stem and progenitor subpopulations within CD34+ve cells during ex vivo erythroid differentiation. Representative double positive FACS plots and graphs are shown from the analysis of CD34+ cells cultured and harvested at the indicated days during ex vivo erythroid differentiation. Cells were stained for human lineage markers (CD3, CD14, CD16, CD19, CD20 and CD56), as well as CD34, CD38, CD90, CD45RA, and CD123. (**A**) FACS analysis of subpopulation of CD34+CD38− (uncommitted progenitors): HSCs (Lin−/CD34+/CD38−/CD45RA−/CD90+); MPPs (Lin−/CD34+/CD38−/CD45RA−/CD90−); and LMPPs (Lin−/CD34+/CD38−/CD90−/CD45RA+) on the indicated days. (**B**) Graph for Figure 7A. (**C**) FACS analysis of subpopulation of CD34+CD38+ (committed-progenitors): CMPs (Lin−/CD34+/CD38+/CD45RA−/CD123+); GMPs (Lin−/CD34+/CD38+ /CD45RA+/CD123+); and MEPs (Lin−/CD34+/CD38+/CD45RA−/CD123−) on indicated days. (**D**) Graph for Figure 7C. Short terms indicate: HSCs: hematopoietic stem cells, MPPs: multipotent progenitors, LMPPs: lymphoid-primed multipotent progenitors, CMPs: common myeloid progenitors, GMPs: granulocyte-macrophage progenitors, MEPs: megakaryocyte erythrocyte progenitors. Error bars indicate mean ± SD of (n = 3) independent experiments conducted on HSPC samples obtained from three healthy individuals.

To kinetically describe and quantify the differentiation potential of diverse HSPC populations towards the erythroid lineage, we performed detailed immunophenotyping of committed and uncommitted progenitors during ex vivo erythroid differentiation. The cells were examined for changes in the expression of cell surface markers using the hematopoietic progenitor marker panel (mentioned in Table 1) every other day until day 12. CD34+ve cells were classified as CD34+CD38− uncommitted progenitors to identify hematopoietic stem cells as HSCs (CD34+CD38−CD90+CD45RA−), MPP (CD34+CD38−CD90+CD45RA−), and LMPP (CD34+CD38−CD90+CD45RA−). The CD34+CD38+ cells were identified as committed progenitors, and they were further sub-gated to identify CMPs (CD34+CD38+CD123+CD45RA−), GMPs (CD34+CD38+CD123+CD45RA+), and MEPs (CD34+CD38+CD123−CD45RA−). The detailed gating strategy adopted is mentioned in Supplementary Figure S2. We observed a mixed population of committed and uncommitted progenitors on day zero (Figure 2A–D). At day zero, HSCs were highest (20.45%) among uncommitted progenitors, followed by MPPs (14.44%) and LMPPs (3.35%). We saw a sharp decline in HSCs by day two and a sustained drop in MPPs until day six. By day three, LMPP had become insignificant (Figure 2A,B). Furthermore, the CMPs (43.25%) were the most abundant of the committed progenitors, while MEPs (3.30%) were the least. CMPs increased steadily until day two then dropped completely by day eight. Similarly, GMPs (14%) were maximum at day zero and gradually decreased during erythroid progression. We additionally observed that MEPs tripled on day two before dropping completely on day eight. (Figure 2C,D). Thus, our finding concludes that CMPs outnumbered MEPs and all progenitors were completely lost by day eight.

### 3.3. Lineage Commitment of Hematopoietic Precursors during Erythroid Differentiation

**Figure 3:**
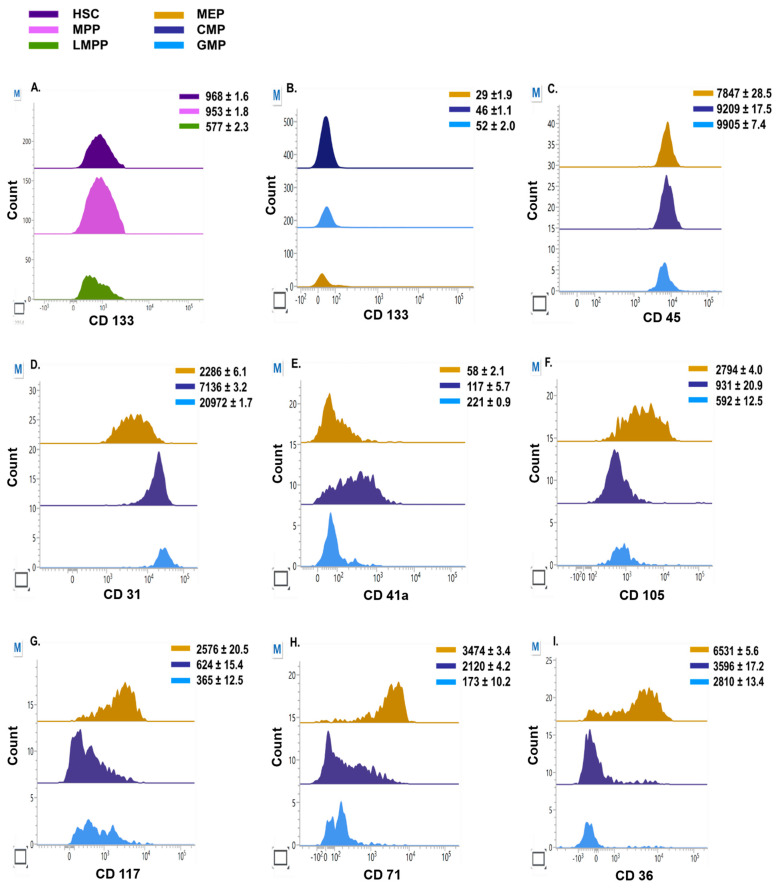
Immunophenotypic expression of surface proteins on progenitors. (**A**,**B**) Median fluorescence intensity (MFI) of CD133 on committed and uncommitted progenitors. (**C**–**I**) MFI of CD45, CD31, CD41a, CD105, CD117, CD71, and CD36 on committed progenitors. The values indicate MFI (+/−SEM) of (n = 3) independent experiments conducted on HSPC samples obtained from three healthy individuals.

Since the lineage commitment of HSPC precursors is still debated [25,26,27], we investigated lineage directionality using the expression pattern of surface markers for each of the progenitor populations. We initially identified CD133 (PROM1), which is well defined in human HSCs [28,29] and observed that its expression was similar in HSCs (MFI 968 ± 1.6) and MPPs (MFI 953 ± 1.8), while LMPPs had the lowest intensity (MFI 577 ± 2.3) (Figure 3A). Furthermore, CD133 was dim in all three committed progenitor populations (Figure 3B). It is reported that erythroid precursors are recognized by low CD45 expression [30,31]; here, we found a similar trend demonstrating that MEPs had the lowest CD45 expression (MFI 7847 ± 28.5) compared with GMPs (MFI 9905 ± 7.4) and CMPs (MFI 9209 ± 17.5) (Figure 3C). CD31 expression has been widely explored in mouse HSCs [32] and erythro-myeloid precursors [33,34]. Therefore, we investigated the expression (MFI) on committed progenitors and identified that CD31 is heterogeneously expressed among the three populations. GMPs highly expressed CD31 (MFI 20972 ± 1.7), followed by CMPs (MFI 7136 ± 3.2) and MEPs (MFI 2286 ± 6.1) (Figure 3D). Furthermore, we looked at the megakaryocytic skewness of erythroid-primed committed progenitors with CD41a and we noticed that they are dimly expressed in these populations, with the maximum intensity seen in GMPs (MFI 221 ± 0.9), followed by CMPs (MFI 117 ± 5.7) and MEPs (MFI 58 ± 2.1) (Figure 3E). Endoglin (CD105) is widely expressed on erythroid progenitors and erythroid-myeloid precursors [35,36] and we found that MEPs had the highest intensity (MFI 2794 ± 4.0) while CMPs and GMPs were comparable with intensities of 931 ± 20.9 and 592 ± 12.5, respectively (Figure 3F). CD117 (c-kit) yielded similar results (Figure 3G). Very bright expressions of erythroid-specific markers, i.e., CD36 and CD71, were observed on MEPs (MFI 6531 ± 5.6, 3474 ± 3.4). CMPs were less bright for CD36 (MFI 3596 ± 17.2) and CD71 (MFI 2120 ± 4.2) than MEPs, while fluorescence intensities of CD36 and CD71 were lowest for GMPs (MFI 2810 ± 13.4, 173 ± 10.2) (Figure 3H,I).

### 3.4. Quantitative Analysis of BFU-E and CFU-E during Ex Vivo Culture

**Figure 4:**
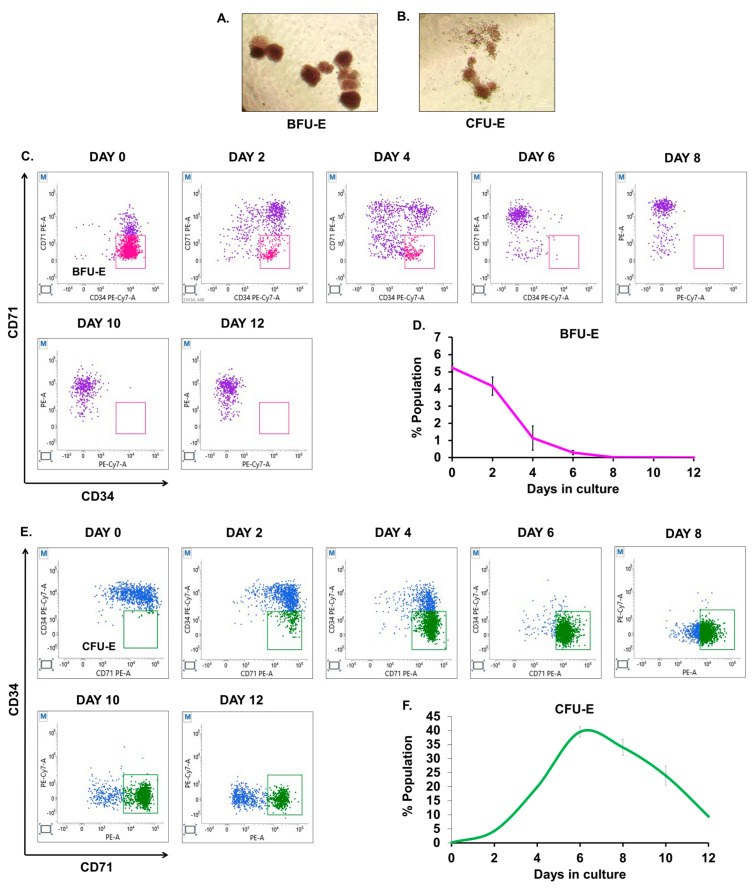

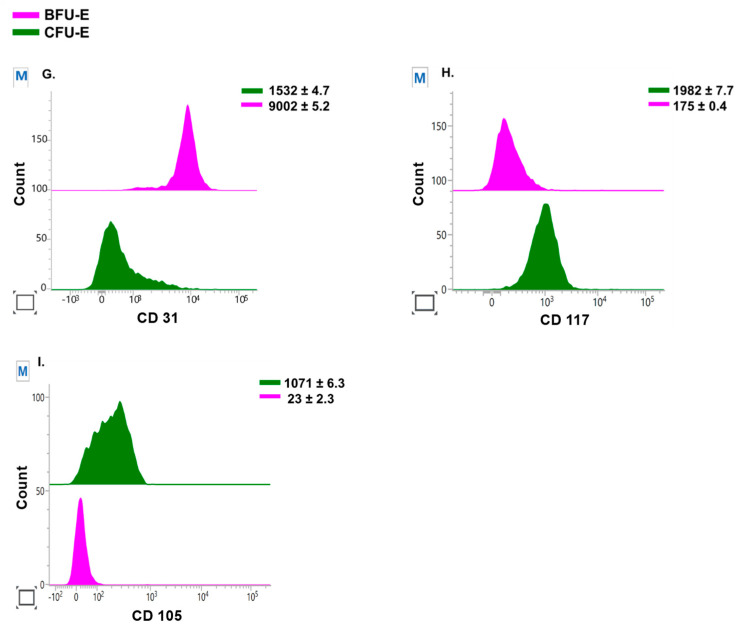
Analysis of BFU-E and CFU-E during ex vivo culture: 10 × 10^4^ CD36−CD34+CD71 low and CD36+CD34−CD71 high cells were sorted from LIN−CD45+GPA−CD123− cells on day two of culture. CD36−CD34+CD71 low cells mostly yielded BFU-E colonies (**A**) and CD36+CD34−CD71 high cells formed CFU-E colonies (**B**) in MethoCult^TM^ (H4434). Colonies were imaged at 4X on day 12 post-seeding the sorted cells. Cultured CD34+ cells were harvested and stained for human lineage markers (CD3, CD14, CD16, CD19, CD20, and CD56) as well as CD45, CD123, GPA, CD36, CD71, and CD34. A total of 20,000 Lin−GPA−CD123−CD45+ cells were sub-gated into CD36−CD34+CD71 low cells as BFU-E cells and CD36+CD34+CD71 high as CFU-E cells, respectively. Representative double-positive FACS plots and graphs depict the populations of (**C**,**D**) BFU-E and (**E**,**F**) CFU-E at indicated time points during ex vivo erythroid differentiation. (**G**–**I**) Median fluorescence intensity (MFI) of CD31, CD117, and CD105 on the population of BFU-E and CFU-E cells. For (**D**,**F**), error bars indicate mean ± SD. For (**G**–**I**), error bars indicate SEM from (n = 3) independent experiments conducted on HSPC samples obtained from three healthy individuals.

The earliest identified erythroid progenitors are BFU-E and CFU-E cells, which have been extensively studied in human bone marrow [15]. We sought to characterize BFU-E and CFU-E cells by sorting and further identifying the dynamic changes occurring in these cells through the application of the BFU-E/CFU-E marker panel (mentioned in Table 2). The detailed gating scheme adopted to assess the progression of BFU-E/CFU-E during erythroid differentiation is described in Supplemental Figure S3. In order to first characterize BFU-E and CFU-E, we sorted CD36−CD34+CD71 low and CD36+CD34−CD71 high cells from the population of LIN−CD45+GPA−CD123− cells on day two of culture. We observed that CD36−CD34+CD71 low cells primarily yielded BFU-E colonies (Figure 4A) while CD36+CD34−CD71 high cells contained CFU-E colonies (Figure 4B) in MethoCult^TM^. To study the dynamic changes in these cells, we harvested cells from our ex vivo erythroid culture on alternate days from day 0 to day 12. A total of 20,000 LIN−CD45+GPA−CD123−cells were sub-gated to look for the population of CD36−CD34+CD71 low cells as BFU-E and CD36+CD34−CD71 high as CFU-E. We identified that BFU-E were maximum at day zero (5.23%), which completely declined at day eight (Figure 4C,D). Likewise, CFU-E gradually started increasing from day two and peaked at day six; it showed 7.5-fold of BFU-E (Figure 4E,F), suggesting that a greater number of mature CFU-E arise from the lesser immature BFU-E. We further examined the expression of CD31, CD117, and CD105 on the above progenitor populations and found that CD31 expression on BFU-Es was the brightest overall (MFI 9002 ± 5.2) whereas expression on CFU-E was weaker (MFI 1532 ± 4.7) (Figure 4G). Moreover, we observed that compared to BFU-E (MFI 175 ± 0.4), CD117 expression was greater in CFU-E (MFI 1982 ± 7.7) (Figure 4H). With CD105, a similar pattern was seen but the expression was noticeably less than that of CD117 (Figure 4I).

### 3.5. Kinetics and Characterization of Terminal Erythroblasts

**Figure 5:**
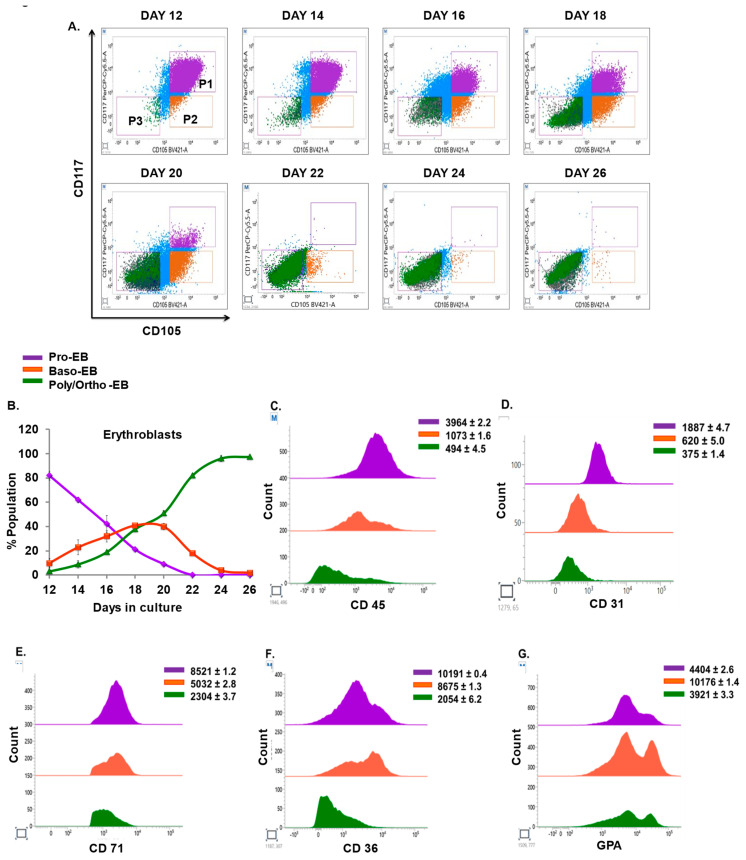

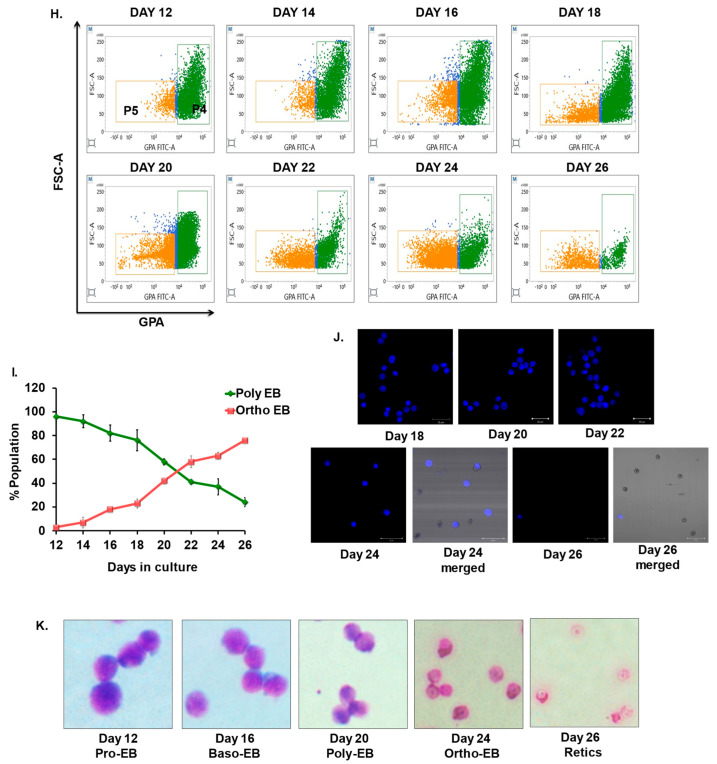
Kinetics and characterization of terminal erythroblasts. A total of 2 × 10^5^ cells in culture were harvested and stained for markers CD45, CD31, CD71, CD36, CD117, CD105, and GPA. (**A**) All erythroid developmental stages were gated as CD71 positive cells. Gated cells were further subdivided into CD105+CD117+ pro-erythroblasts (Pro-EB (P1)), CD105+CD117− basophilic erythroblasts (Baso-EB (P2)) and polychromatophilic erythroblasts and orthochromatophilic erythroblasts (Poly/Ortho-EB) in the CD105−CD117− (Poly-Ortho EB (P3)) region. (**B**) Representative graph for Figure 7A. (**C**–**G**) Median fluorescence intensity (MFI) of CD45, CD31, CD71, CD36, and GPA on the population of Pro-EB, Baso-EB, and Poly/Ortho EB. (**H**) Poly- and orthochromatic erythroblasts (Poly/Ortho-EB) in the P3 gate were further discriminated based on the surface expression of GPA and FSC. GPA-high FSC high cells are gated as Poly-EB (P4) while GPA dim/intermediate and FSC low cells are identified as Ortho-EB (P5). (**I**) Representative graph for Figure 5H. (**J**) Analysis of enucleation in erythroid cells at indicated days by Hoechst staining. (**K**) Morphological analysis of erythroid cells at indicated days by May–Grunwald–Giemsa staining. For (**B**,**I**), error bars indicate mean ± SD. For (**C**–**G**), error bars indicate SEM from (n = 3) independent experiments conducted on HSPC samples obtained from three healthy individuals.

In our study, distinct expression of CD71, CD117, and CD105 is first observed at the MEP stage followed by CFU-E; hence, we used these three markers to define the terminal stages of erythropoiesis. CD71 steadily declines in the later stages (as observed in Figure 1F); therefore, we employed CD71 as a gating marker to differentiate the later stages of erythroid differentiation. The expression of CD117 lost at the basophilic stage (Baso-EB) and that of CD105 is lost at the poly/orthochromatic (Poly/Ortho-EB) stage [17]; therefore, we identified Pro-EB as CD71+CD117+105+ cells (P1), Baso-EB as CD71+CD117+105− (P2) cells, and Poly/Ortho EB as CD71+CD117−105− (P3) cells from CD71 positive cells (Figure S4). As cells progressed toward the terminal stages of differentiation, we observed a gradual decrease in Pro-EB and a subtle rise in Baso-EB and Poly/Ortho-EB. We found that the population of Baso-EB peaked on day 19 of differentiation and then continued to fall, whereas Poly/Ortho-EB started to appear on day 14 and peaked on day 24 of differentiation (Figure 5A,B). To evaluate the expression of specific markers (CD45, CD31, CD71, CD36, and GPA) on subsets of Pro-EB, Baso-EB, and Poly/Ortho-EB, we examined the MFI of these markers on each subset of cells. We discovered that Pro-EB (MFI 3964 ± 2.2) had the highest CD45 intensity, followed by Baso-EB (MFI 1073 ± 1.6) and Poly/Ortho-EB (MFI 494 ± 4.5) (Figure 5C). We further noticed a reduction in the expression of CD31, which varied from 2-1.3-fold on the population of Pro-EB, Baso-EB, and Poly/Ortho-EB (Figure 5D). Moreover, we observed that Pro-EB expressed more CD36 (MFI 10,191 ± 0.4) than CD71 (MFI 8521 ± 1.2). For Baso-EB, a similar pattern was seen. Furthermore, we observed that the expression of CD71 (MFI 2304 ± 3.7) and CD36 (MFI 2054 ± 6.2) on Poly/Ortho-EB was nearly identical (Figure 5E,F). Later, we discovered that GPA peaked at the basophilic stage (MFI 10,176 ± 1.4) (Figure 5G) which is in concordance with previous studies on regenerating human bone marrow [17]. As a result, we made a distinction between the Poly/Ortho-EB population based on the forward scatter property and GPA expression. Polychromatic erythroblasts (Poly-EB (P4)) were defined as cells with higher FSC and GPA intensity, whereas orthochromatic erythroblasts (Ortho EB (P5)) were defined as cells with low FSC and intermediate/low GPA expression. Based on the gating scheme, we noticed that as cells matured during erythroid differentiation, Poly-EB gradually decreased, and Ortho EB eventually increased (Figure 5H,I). We further analyzed the enucleation in maturing erythroblasts and observed that the cells underwent complete enucleation by day 26 (Figure 5J). Morphological analysis by May–Grunwald–Giemsa further validated the physiological transition in erythroid cells (Figure 5K).

### 3.6. NSD1 Knockdown Alters Early Human Erythroid Progenitors

**Figure 6:**
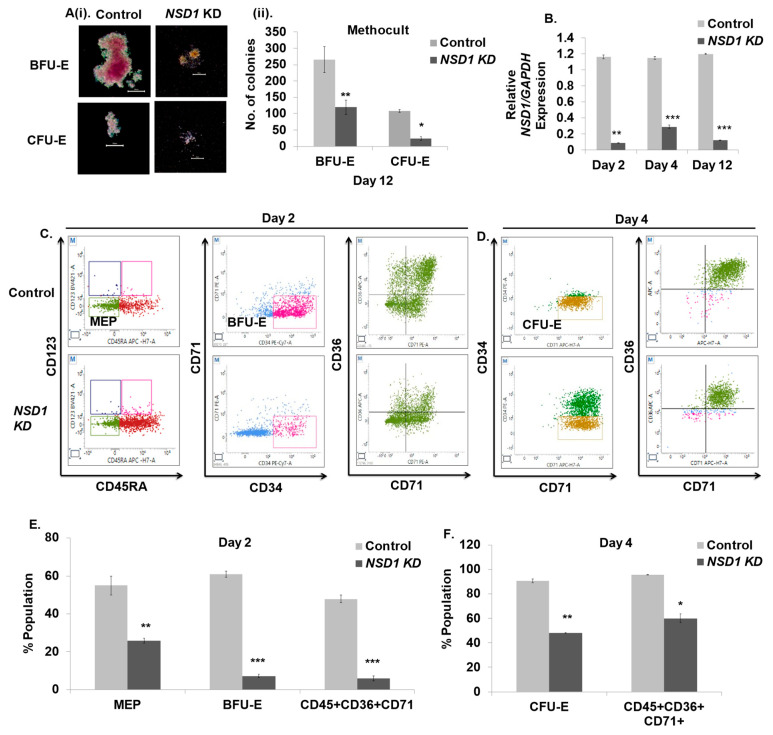
*NSD1* knockdown impairs early human erythroid differentiation. Cells in culture were transduced with lentivirus containing pLKO.1 scrambled (control) or NSD1 shRNA at days 2, 4, and 12 of differentiation. NSD1 transduced cells were analyzed after 72 h for knockdown induction and compared to control. (**A**(**i**)) Representative images of colonies formed in MethoCult^TM^ (H4434) upon NSD1 KD at day zero (post cytokine induction overnight). After 24 h of infection, 10 × 10^4^ were seeded in MethoCult^TM^ (H4434) containing a recombinant cytokine cocktail of SCF, IL-3, EPO, and GM-CSF. (**ii**) No. of colonies formed upon *NSD1* knockdown. (**B**) Relative *NSD1* mRNA expression with *GAPDH* as an internal control upon *NSD1* KD at days 2, 4, and 12 of culture. (**C**) *NSD1* KD cells were analyzed using markers (listed in Table 1 and Table 2) at the MEP and BFU-E stage on day two. A total of 20,000 cells were gated to identify the population of MEPs as Lin−/CD34+/CD38+/CD45RA−/CD123− cells and the population of BFU-E as (LIN−CD45+CD123−GPA−CD36−CD34+CD71low) cells by flowcytometry. Double-positive CD36+CD71+cells were also analyzed. (**D**) Analysis of CFU-E upon *NSD1* KD at day four. A total of 20,000 cells were gated in the LIN−CD45+CD123−GPA− population to identify CFU-E as CD36+CD34−CD71 high cells. Double-positive CD36+CD71+cells were also analyzed. (**E**,**F**) Representative graphs for the Figure 7C,D. Error bars indicate mean ± SD of (n = 3) independent experiments conducted on HSPC samples obtained from three healthy individual donors. * *p* < 0.05, ** *p* < 0.005, *** *p* < 0.0005.

Inactivation of *NSD1* has been shown to induce erythroleukemia in mice, yet its role in human erythropoiesis is unexplored. Having established the approach to quantitatively monitor ex vivo erythroid differentiation, we explored whether the application of this method can be used to study the role of transcription/epigenetic factors at different stages of erythropoiesis. Hence, to study the role of *NSD1,* we employed a shRNA-targeted lentiviral-mediated knockdown (KD) approach. We first undertook the KD at day zero (post cytokine induction overnight) and seeded 10,000 cells in MethoCult^TM^ to observe colony formation. On day 12 we observed a significant reduction in BFU-E and CFU-E colonies with distorted morphology, as previously reported in mice [20] (Figure 6A(i,ii)). Therefore, we performed the KD of *NSD1* on days 2, 4, and 12 of erythroid differentiation. (Figure 6B) shows a 70–90% knockdown efficiency by two independent shRNAs after three days of KD induction at the three timepoints. We observed the effect of the *NSD1* KD on MEPs and found that they were 57–58% reduced upon *NSD1* KD at day two (Figure 6C,E). Subsequently, we noted an 80% reduction in BFU-E and a 30% reduction in CFU-E upon *NSD1* KD on days two and four (Figure 6C,E,F). Furthermore, a significant reduction in double positive CD45+CD36+CD71+ cells was also reported (Figure 6C–F). Thus, the above data suggest that *NSD1* attenuates early human erythroid progenitors.

### 3.7. NSD1 Knockdown Blocks Terminal Differentiation in Maturing Erythroblasts

**Figure 7:**
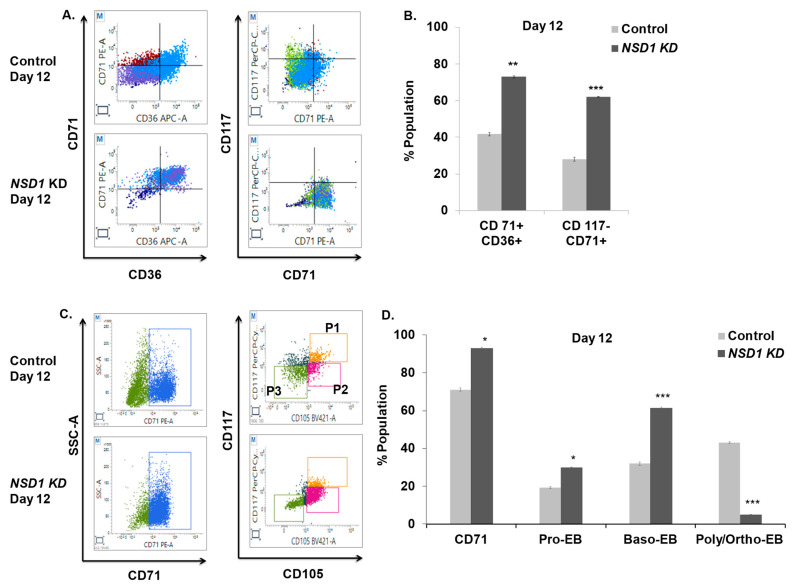
*NSD1* knockdown leads to a maturation block in terminally differentiating erythroblasts. Representative FACS plots and bar graphs are shown from the analysis of *NSD1* knockdown cells using an shRNA-mediated knockdown approach on day 12 to see the effect on terminally differentiating erythroblasts. (**A**) Representative FACS plots for dual-stained markers CD36+CD71+ and CD117+CD71+. (**B**) Bar plot for Figure 7A. (**C**) A total of 20,000 CD71 cells were gated to identify CD71+CD105+CD117+ cells as (Pro-EB (P1)), CD71+CD105+CD117− cells as (Baso-EB (P2)), and (Poly/Ortho EB) as CD71+CD105−CD117−(P3) cells. (**D**) Bar plot for Figure 7C. Error bars indicate mean ± SD of (n = 3) independent experiments conducted on HSPC samples obtained from three healthy individual donors. * *p* < 0.05, ** *p* < 0.005, *** *p* < 0.0005.

As shown above, the KD of *NSD1* alters early erythroid progenitors, but its involvement in terminal human erythroid differentiation is unknown. Using the KD approach outlined above, we evaluated the effect on terminally differentiating erythroblasts using an erythroid panel (mentioned in Table 3). On day 12 after 72 h of KD, we noted double-positive cells CD36+CD71+ were considerably increased (almost two-fold) relative to the control. We also observed an increase in the number of CD117−CD71+ cells (cells in between CFU-E and proerythroblast stage) (Figure 7A,B). Using CD71 as a gating marker, we studied terminal erythroid differentiation and observed that total CD71 expression showed a 1.3-fold increase in KD cells. Moreover, we noted Pro-EB increased 1.5-fold and Baso-EB increased 1.9-fold, while terminally differentiating erythroblasts, i.e., Poly/Ortho EB, were reduced 8.6-fold compared with the control, suggesting a maturation block in these cells (Figure 7C,D). This finding was further validated with reduced expression of *β-globin* gene expression upon KD of *NSD1* at day 12. (Figure S6). The above results are in concordance with data shown in mice studies [20] and hence signify that *NSD1* KD leads to a maturation block in terminally differentiating human erythroblasts.

### 3.8. MDS and PRCA Patients Display Altered Erythropoiesis

**Figure 8:**
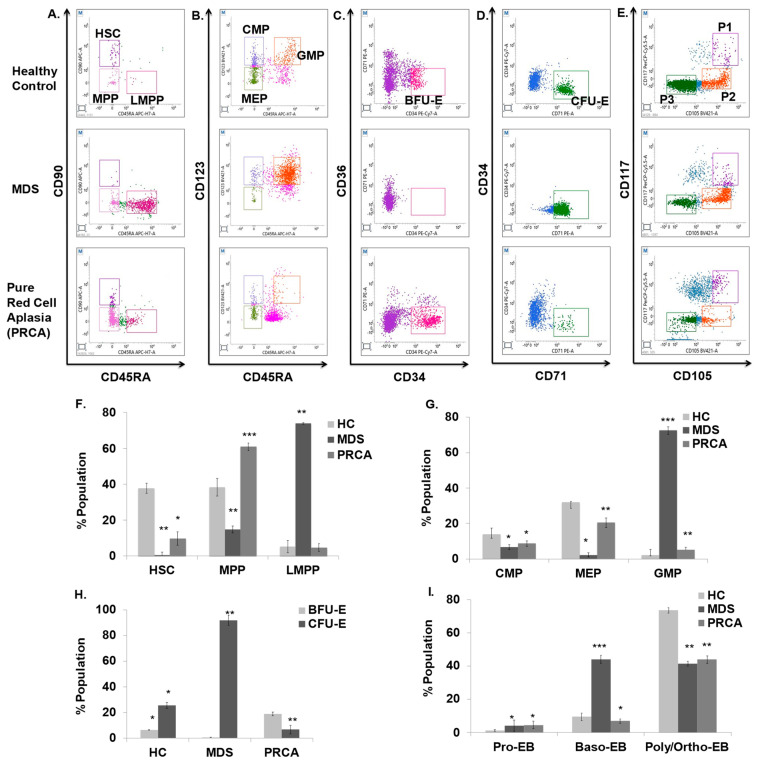
FACS analysis of bone marrow profiles of MDS and pure red cell aplasia (PRCA) patients. Dot plots from the analysis of bone marrow aspirates to identify (**A**) uncommitted progenitors (HSCs, MPPs, LMPPs) and (**B**) committed progenitors (CMPs, GMPs, MEPs), (**C**,**D**) BFU-E and CFU-E populations, (**E**) Pro-EB (P1), Baso-EB (P2) and Poly-Ortho EB (P3) in MDS and PRCA patients compared with the healthy control (HC). (**F**,**G**) Representative bar graph for Figure 7A,B. (**H**) Representative bar graph for Figure 7C,D. (**I**) Representative bar graph for Figure 7E. Error bars indicate mean ± SD of (n = 3) independent experiments conducted on BM aspirates of three healthy individuals or three patient samples. * *p* < 0.05, ** *p* < 0.005, *** *p* < 0.0005.

MDS and PRCA patients represent clonal disorders of ineffective hematopoiesis that finally give rise to altered erythropoiesis [37,38]. Currently, the methods for identifying stage-specific erythroid dyspoiesis remain limited [14,39,40,41]. Therefore, we tested the applicability of our marker panels to monitor erythroid differentiation in normal human bone marrow (BM), as well as in patients affected with MDS (erythroid dysplasia) and PRCA. We noted that among uncommitted progenitors, the HSC population was 40% in the control group, which was significantly less than 10% in PRCA, and an almost negligible population in MDS. MPPs were highest in PRCA (61%), while MDS had reduced expression (15%) compared with the control (39%). LMPPs were highest in the MDS subgroup (74%), while PRCA had an almost similar expression as the control (Figure 8A,F). Among committed progenitors, CMPs from MDS and PRCA were 58–63% reduced compared with the healthy control (14%). GMPs were 3.5 times higher in MDS compared with the healthy control (22%). The MEPs were over 30% in the healthy group, with an absence in MDS and a reduced population (20%) in PRCA (Figure 8B,G). The BFU-E were >6.5% in the healthy control, followed by increased numbers (19%) in PRCA and complete loss in MDS patients (Figure 8C,H). Further mature progenitors CFU-E in MDS were nearly four-fold compared with the healthy control (25%), while PRCA displayed reduced expression of CFU-E (6%) (Figure 8D,H). To examine terminal erythropoiesis, we noted redundancy in Pro-EB and Baso-EB in MDS, while in PRCA redundancy was only noted in Pro-EB. Baso-EB were slightly reduced in numbers compared with the healthy control. Poly- and orthochromatic stages were significantly decreased in patients showing a block in the maturation stages (Figure 8E,I). Morphological features of bone marrow aspiration smears using May–Grunwald–Giemsa staining is shown in Figure S7. Thus, the above results signify that the approach can be used to decipher the abnormalities occurring in patients with erythroid dyspoiesis.

## 4. Discussion

Adult human erythropoiesis is a complex process that begins in bone marrow with the commitment of hematopoietic stem cells to the erythroid lineage precursors, which finally mature to form red blood cells. The transition gives rise to distinct development stages with each round of cell division. In the current study, we developed a strategy to study erythroid differentiation from HSPCs by using marker panels to study stage-specific kinetics from uncommitted/committed precursors to terminally differentiated erythroblasts. All stages were studied during the ex vivo culture of HSPCs from days 0 to 26. We analyzed hematopoietic and erythroid precursor stages from days 0 to 12, while terminal stages were studied from days 12 to 26 of differentiation. At a molecular level, this continuum was validated with a decrease in *GATA2,* which initiates megakaryocytic switching [42], and an increase in the expression of erythroid specific genes, i.e., *KLF1, GATA1,* and *β*-globin genes that mark the process of erythroid differentiation and are reported in previous studies [12].

Analysis of hematopoietic precursors during erythroid differentiation revealed that at day zero, mixed populations of uncommitted (HSCs, MPPs, LMPPs) and committed (GMPs, CMPs, MEPs) progenitors were observed (Figure 2B,D). Our findings highlight that these progenitors exist as discrete precursors in a culture. These findings are in line with the study by Notta et al., who demonstrated that the blood hierarchy predominantly consists of multipotent cells, such as HSCs and MPPs, as well as unipotent progenitors. These unipotent progenitors primarily contain myeloid, erythroid, and megakaryocytic potential in the bone marrow [43]. We noticed that CMPs make up the majority of HSPCs, followed by GMPs, HSCs, MPPs, MEPs, and LMPPs, and only MEPs increased 3.1-fold, whereas most populations were lost by day eight (Figure 2D). The lineage potential of the precursor stages to preferentially differentiate into erythroid lineage is well anticipated by many studies [25,44]. One such study by Notta et al. identified GATA-1-positive MEPs as being derived from multipotent cells, while CMPs are heterogeneous and multilineage, containing myeloid, erythroid, and megakaryocytic potential [43]. Another study by Mori et al. concluded that erythroid development culminates from CD71−CD105− CMPs via CD71+CD105 CMPs and CD71+CD105 MEPs to erythroid progenitors. In the current study, we found that MEPs had the highest expression of CD36, followed by CD71, CD105, CD117, CD31, and CD41a, whereas CMPs had the maximum expression of CD31, followed by CD36, CD71, CD105, CD117, and CD41a. For GMPs, we found that the expression of CD31 was the brightest, followed by CD105, CD117, CD41a, and CD71. We observed that GMPs were biased toward the megakaryocytic lineage (correlated with higher expression of CD41a versus CD71 expression) and that MEPs had a higher proliferative potential than CMPs. Mori et al. reported that the expression of CD36 was very low in CMPs; however, we found that CMPs expressed CD36 at considerably higher levels [45]. We further found that MEPs express CD117, CD105, and CD71 at higher levels than CMPs. Our finding that GMPs had the highest expression of CD31 coincides with the fact that GMPs are direct precursors of multi-lymphoid progenitors (MLPs), which in turn give rise to the myelomonocytic lineage from which the megakaryocyte and erythroid arm originates [43].

Li et al. identified BFU-E as CD45+GPA−CD123−CD34+CD36−CD71 low cells and CFU-E as CD45+GPA−CD123−CD34+CD36−CD71 high cells [15]. The dynamics of the transition from BFU-E to CFU-E have not yet been documented well; thus, we deciphered the erythroid transition from day 0 to day 12 of the ex vivo culture and found that BFU-E (5.23%) occupied a major proportion in G-CSF-mobilized peripheral blood than CFU-Es (0.09%) per 20,000 LIN−CD45+GPA−CD123− cells. On day six, we found that CFU-E were 7.5-fold of BFU-E and reported that the proliferative potential of BFU-E is more than CFU-E (Figure 4D). We further sought to identify other probable markers that would aid in more precisely distinguishing BFU-E from CFU-E. We found that the expression of CD31 was highest in BFU-E, followed by CD117 and CD105. We next observed that CFU-E had a maximum intensity of CD117, followed by CD31 and 105 (Figure 4F–I). Since CD31 is widely expressed on erythro-myloid progenitors [32], and it has been recently identified that a subset of CD45 low/high CD34+ve cells gives rise to GPA+ve erythroblasts [19], this marker, along with CD117 (as a marker of responsiveness to SCF), could further help in refining the strategy to isolate more refined BFU-E from (GPA−CD123−) erythroid compartments in the future. Yan et al. redefined the continuum based on CD105 and GPA profiles [14], but in the current study, we identified that CD105 was considerably expressed at lower levels in BFU-E.

Terminal erythroid differentiation begins when CFU-E progresses into Pro-EB, which eventually matures into Poly/Ortho-EB and gives rise to mature RBCs. Utilizing the CD71 gating marker, we immunophenotyped Pro-EB as CD71+CD117+CD105+ cells, Baso-EB as CD71+CD117−CD105+ cells, and Poly/Ortho EB as CD71+CD117−CD105− cells (Figure 5A,B). Our findings, which are consistent with those of previous investigations, suggest that the expression of GPA is maximum at the basophilic phase [17]; however, other studies report GPA to be maximum at the end stages of erythropoiesis [45]. The expression of CD36 is found to be maximum at the basophilic stage [17]; however, we found that the expression of CD36 progressively decreased from Pro-EB to Poly/Ortho EB as in the case of CD71. We first report the progressive decrease in CD31 expression from Pro-EB to Poly/Ortho EB and that the expression of CD31 was similar to CD45 expression. Further, based on the expression of GPA and FSC, we were able to discriminate Poly/Ortho-EB as FSC high/GPA high and FSC low/GPA intermediate/low cells.

*NSD1* belongs to the SET domain containing the NSD family of histone lysine methyltransferases (HKMTs), which have been implicated in human development and malignancies [46]. A recent study in mice [20] confers its important role in erythroleukemia, although its function in human erythropoiesis has yet to be investigated. As a result, we used ex vivo erythroid differentiation to reveal its significance in human erythropoiesis, employing marker panels in the current investigation. *NSD1* knockout studies in mice have shown no significant changes in the population of MEPs and CFU-E; we present that the population of MEPs and BFU-E were substantially decreased upon *NSD1* KD at day two, whereas KD at day four resulted in a reduction in CFU-E (Figure 6E,F). These findings are consistent with our MethoCult^TM^ data which show erythroid colony depletion. Additionally, to investigate the function of terminally differentiating erythroid cells, we discovered an increase in the number of cells (CD117−CD71+) that give rise to Pro-EB. Similarly, Pro-EB and Baso-EB increased significantly with a decrease in Poly/Ortho-EB (Figure 7B,D). Our findings indicate that *NSD1* plays an important role in both the early and late phases of erythroid differentiation; hence, these marker panels may be utilized to analyze stage-specific alterations during erythroid development in knockdown investigations, aiming to study the role of specific transcription /epigenetic factors regulating erythropoiesis.

MDS and PRCA represent the diseases of inefficient erythropoiesis [37,38] and have been classically defined by the increased coefficient of variation (CV) of CD36 and CD71 expression on the erythroid precursor. Previous studies have highlighted the same context with little focus on stage-specific aberrancies [31,41]. Recently, Yan et al. broadly identified defects in an MDS group of patients with focus on erythroid stages [14]; however, in this research we studied all stages, from HSPCs to terminal stages, of erythroid differentiation and show that at progenitor stages, HSCs, MPPs, and BFU-E were severely decreased, with elevated levels of LMPPs, GMPs, and CFU-E in the MDS group. A subtle decrease in HSCs, MEPs, and CFU-E and an increase in populations of MPPs and BFU-E was noted in the PRCA group. Similarly, at the terminal stages we reported a maturation block in both groups, with elevated Baso-EB in the MDS group. Hence, utilizing our panels, we were able to decipher the defects in the hematopoietic, erythroid progenitor, and terminal stages in these patients by examining the bone marrow. By utilizing our marker panels in such patients, stage-specific anomalies due to erythroid dyspoiesis may be detected, which can be helpful in defining treatment protocols for these patients.

## 5. Conclusions

Overall, our study encompasses a complete characterization of well-known stages from HSPCs to RBCs. The marker panels can be helpful for the identification of defects arising from disordered erythropoiesis and can also be used to study the effect of gene alterations on cellular phenotypic properties during normal erythropoiesis. Furthermore, a FACS analysis of progenitor stages using these marker panels followed by sorting of specific cell population can be used to study the mechanistic insights at the molecular level during normal and defective erythropoiesis. Thus, we propose that our immunophenotypic strategy will serve as a helpful tool for advancing our comprehensive understanding of normal and defective human erythropoiesis.

## Figures and Tables

**Table 1:** Hematopoietic progenitor marker panel.

Antibody	Source	Catalogue Number
APC-R700 Mouse anti-Human CD45	BD Horizon^TM^	566042
PE Mouse anti-Human CD117	BD Biosciences^TM^	555714
Brilliant Violet 605^TM^ Mouse anti-Human CD38	BioLegend^TM^	356642
PE-Cy7^TM^ Mouse anti-Human CD34	BD Biosciences^TM^	348791
Brilliant Violet 510^TM^ anti-human Lineage Cocktail (CD3, CD14, CD16, CD19, CD20, CD56)	BioLegend^TM^	348807
APC Mouse anti-Human CD90	BD Biosciences^TM^	559869
APC-H7 Mouse anti-Human CD45RA	BD Biosciences^TM^	560674
Brilliant Violet 421^TM^ Mouse anti-Human CD123	BioLegend^TM^	306018

**Table 2:** BFU-E/CFU-E marker panel.

Antibody	Source	Catalogue Number
APC-R700 Mouse anti-Human CD45	BD Horizon^TM^	566042
FITC anti-Human Lineage cocktail1 (Lin1) (CD3, CD14, CD16, CD19, CD20, CD56)	BD FastImmune^TM^	340546
CD235a (Glycophorin A) Mouse anti-Human Super bright 600	eBioscience^TM^	63-9987-41
Brilliant Violet 421^TM^ Mouse anti-Human CD123	BioLegend^TM^	306018
APC Mouse anti-Human CD36	BD Pharmingen^TM^	550956
PE-Cy7^TM^ Mouse anti-Human CD34	BD Biosciences^TM^	348791
PE Mouse anti-Human CD 71	BD Pharmingen^TM^	555537

**Table 3:** Erythroid marker panel.

Antibody	Source	Catalogue Number
PE Mouse anti-Human CD 71	BD Pharmingen^TM^	555537
PE-Cyanine 5 Mouse anti-Human CD 117	eBioscience^TM^	15-1178-41
Brilliant violet 421^TM^ anti-Human CD105	BioLegend^TM^	323219
FITC Mouse anti-Human CD235a	BD Pharmingen^TM^	559943

**Table 4:** Sequence of oligos used for cloning.

Oligos	Sequence
hTRC Scrambled	Forward 5′-CCGGCCGCAGGTATGCACGCGTCTCGAGACGCGTGCATACCTGCGGTTTTTG-3′ Reverse 5′-GGCGTCCATACGTGCGCAGAGCTCTGCGCACGTATGGACGCCAAAAACTTAA-3′
h*NSD1* CDS	Forward 5′-CCGGTCCAGTGAGAACTCGTTAATACTCGAGTATTAACGAGTTCTCACTGGATTTTTG-3′ Reverse 5′-AATTCAAAAATCCAGTGAGAACTCGTTAATACTCGAGTATTAACGAGTTCTCACTGGA-3′
h*NSD1* UTR	Forward 5′-CCGGGTGCTAATTTCACGGTATAAACTCGAGTTTATACCGTGAAATTAGCACTTTTTG-3′ Reverse 5′-AATTCAAAAAGTGCTAATTTCACGGTATAAACTCGAGTTTATACCGTGAAATTAGCAC-3′

**Table 5:** List of SYBR green primers used in RT-qPCR.

Target	Sequence
h*NSD1*	For: 5′AGG TAC AGG AGC AGG TGC ACA-3′
	Rev: 5′AGC ACT AGA TCG ACC TCG GGC-3′
h*GAPDH*	For: 5′GTGGTCTCCCTGACTTTCAACAGC-3′
	Rev: 5′A TGAGGTCCACCTGCTTGCTG-3′

## Data Availability

Not applicable.

## Supplementary Materials

The following supporting information can be downloaded at: https://www.mdpi.com/article/10.3390/cells12091303/s1, Figure S1: Changes in the expression of human erythroid genes during ex vivo erythroid culture. Figure S2: Hematopoietic marker panel gating strategy. Figure S3: BFU-E/CFU-E marker panel gating strategy. Figure S4: Time course of CD71 expression during ex vivo human erythroid differentiation. Figure S5: Erythroid panel gating strategy. Figure S6: KD of *NSD1* affects *β-globin* gene expression. Figure S7: MGG morphology of bone marrow smears of HC versus MDS/PRCA patients. Table S1: List of TaqMan Probes and Primers/SYBR Green Primers used in RT-qPCR. Table S2: List of other antibodies used.
