# Supplementary material for: Analysis of Immunophenotypic Changes during Ex Vivo Human Erythropoiesis and Its Application in the Study of Normal and Defective Erythropoiesis
Source: Cells. 2023 May 2;12(9):1303. doi: 10.3390/cells12091303 (PMC10177526; doi:10.3390/cells12091303)
Supplement: Supplementary file 1 [file cells-12-01303-s001.zip › cells-2329875-supplementary.pdf]

cells MDPI

Supplemental Information

Analysis of Immunophenotypic Changes During Ex Vivo Human Erythropoiesis and Its Application in the Study of Normal and Defective Erythropoiesis

Shobhita Katiyar [1,†], Arunim Shah [1,†], Khaliqur Rahman [2], Naresh Kumar Tripathy [2], Rajesh Kashyap [2], Soniya Nityanand [2] and Chandra Prakash Chaturvedi [1,*]

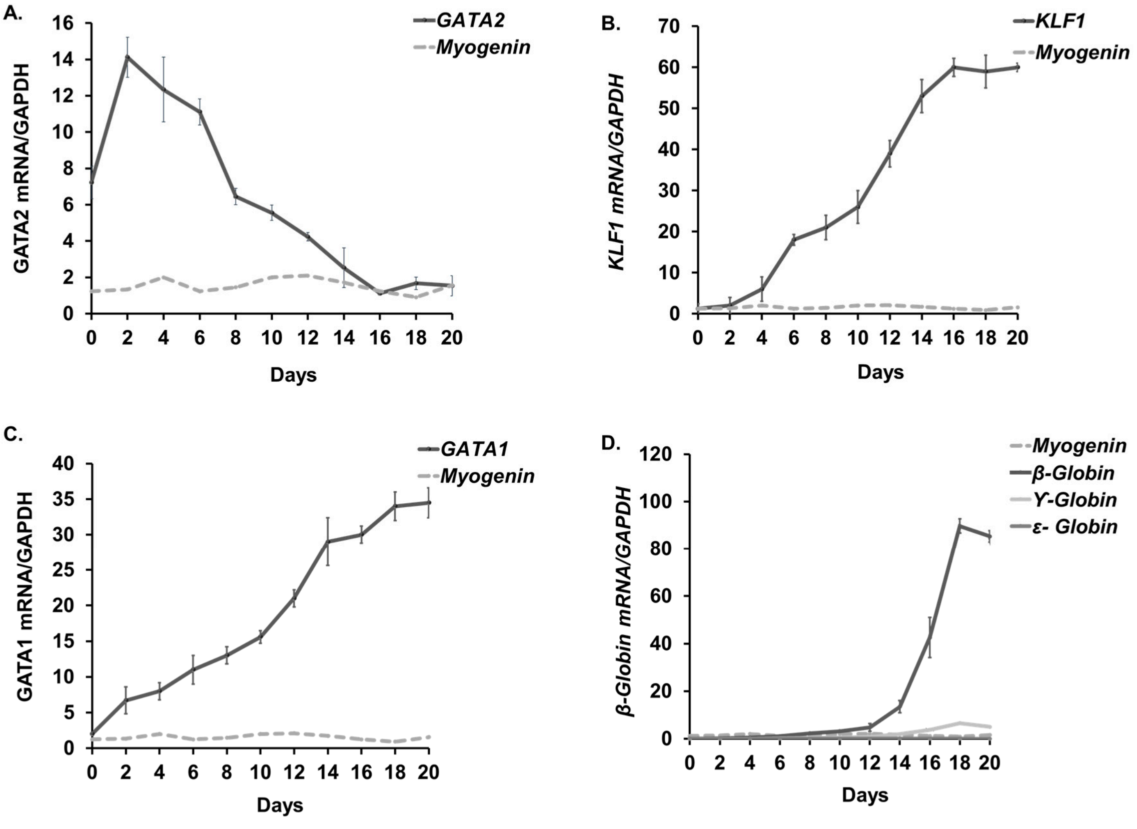

Figure S1: Changes in the expression of human erythroid genes during *ex-vivo* erythroid culture. (A) Relative expression of *GATA2* gene (B) Relative expression of KLF*1* gene (C) Relative expression of GATA*1* gene (D) Relative expression of *β,γ,ε globin* genes, with respect to *GAPDH*. *Myogenin* is taken as a negative control. Error bars indicate mean ± SD of (n=3) independent experiments conducted on HSPC samples obtained from three healthy individual doners.

.

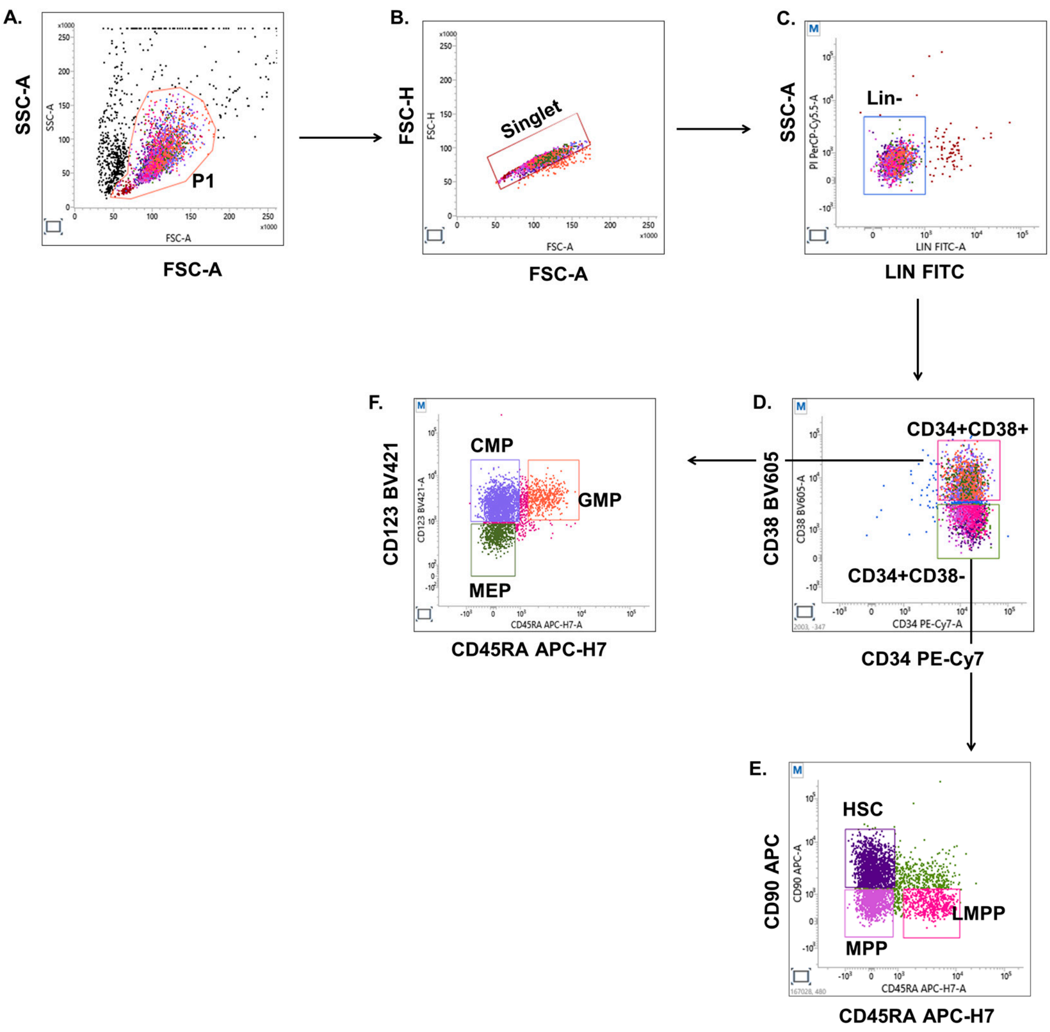

Figure S2: Hematopoietic marker panel gating strategy.

Representative FACS plots are shown from the analysis of cultured hematopoietic stem cells (CD34+ve) isolated from G-CSF-mobilized peripheral blood from a healthy donor, with overnight cytokine induction. Harvested cells were stained for human lineage markers (CD3, CD14, CD16, CD19, CD20 and CD56), as well as CD34, CD38, CD90, CD45RA, and CD123. The gating strategy identifies cells in an (A) forward/side scatter plot (FCS-A and SSC-A) and then single cells are discriminated from doublets using FSC-A/FSC-H plot (B). (C) LIN-cells are gated to identify (D) uncommitted progenitors (CD34+CD38-) and committed progenitors (CD34+CD38+). (E) Uncommitted progenitors are gated to identify; HSCs (Lin-/CD34+/CD38-/CD45RA-/CD90+); MPPs (Lin-/CD34+/CD38-/CD45RA-/CD90-); and LMPPs (Lin-/CD34+/CD38-/CD90-/CD45RA+) (F) Committed progenitors are gated to identify; CMPs (Lin-/CD34+/CD38+/CD45RA-/CD123+), GMPs (Lin/CD34+/CD38+ /CD45RA+/CD123+), and MEPs (Lin-/CD34+/CD38+/CD45RA-/CD123-). Short terms indicate; HSCs: Hematopoietic stem cells, MPPs: Multipotent progenitors, LMPPs: Lymphoid-primed multipotent progenitors, CMPs: Common myeloid progenitors, GMPs: Granulocyte-macrophage progenitors, MEPs: Megakaryocyte-erythrocyte progenitors.

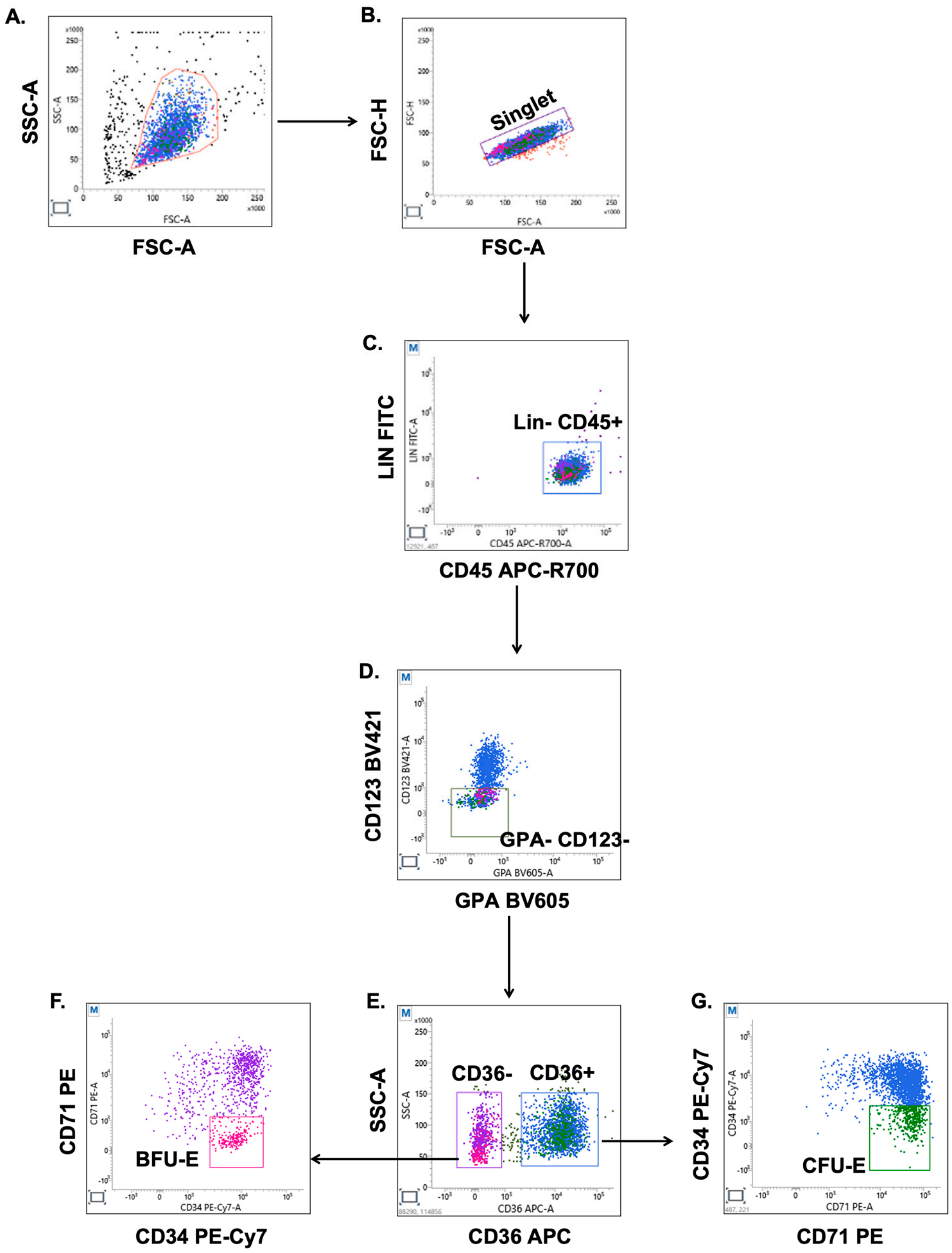

Figure S3: BFU-E/CFU-E marker panel gating strategy

Representative FACS plots are shown from the analysis of cells harvested on day 2 of erythroid culture. Harvested cells were stained for human lineage markers (CD3, CD14, CD16, CD19, CD20, and CD56), as well as CD45, CD123, GPA, CD36, CD71, and CD34. The gating strategy identifies cells in (A) forward/side scatter plot (FCS-A and SSC-A) and then single cells are discriminated from doublets using FSC-A/FSC-H plots (B). (C) LIN-CD45+ cells are gated to identify (D) GPA-CD123- cells. These cells were sub-gated into (E) CD36- and CD36+ cells. CD36- cells are further sub-gated to identify (F) CD34+CD71low cells as BFU-E and (G) CD36+CD34+CD71 high cells as CFU-E, respectively.

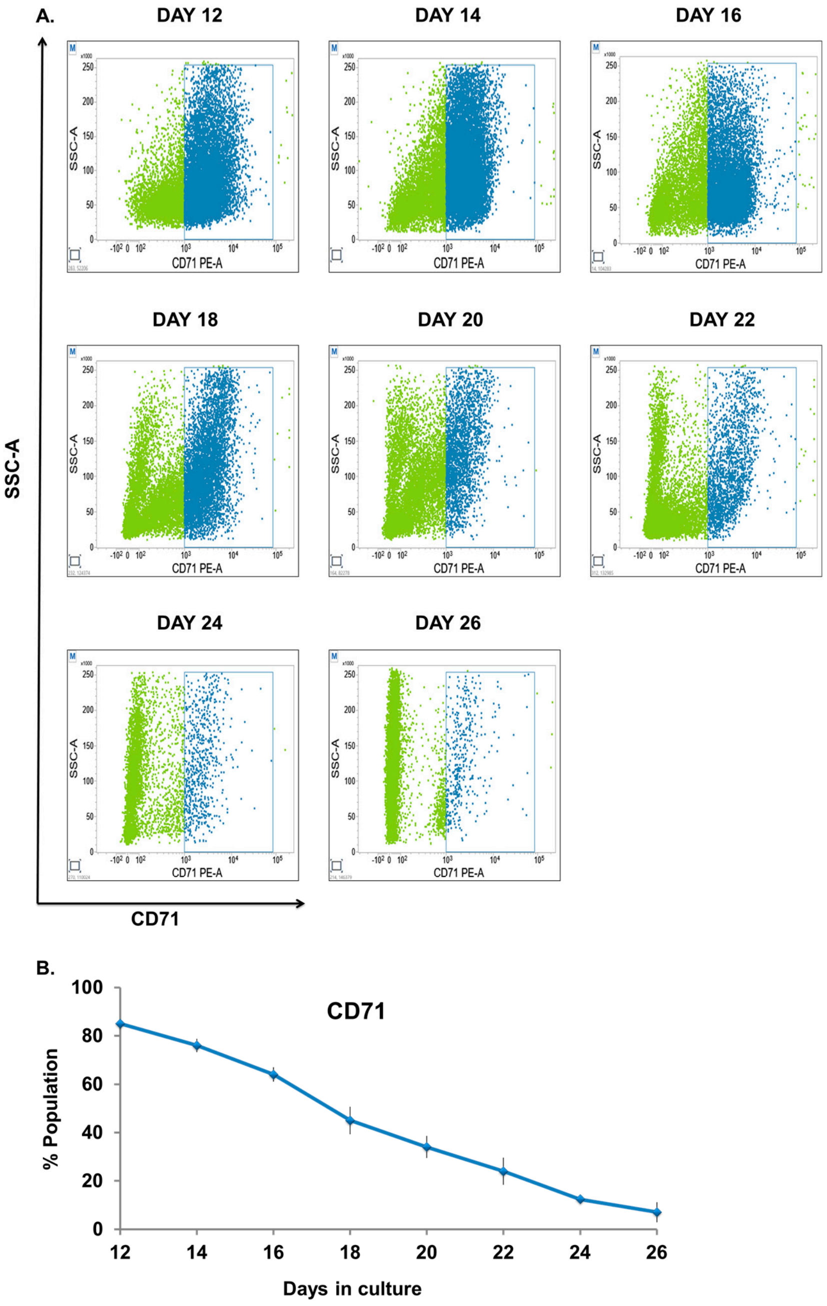

Figure S4: Time course of CD71 expression during ex-vivo human erythroid differentiation.
(A) Representative FACS plots and (B) graph are shown from the analysis of harvested cells at the indicated days during *ex-vivo* erythroid differentiation. Error bars indicate mean ± SD of (n=3) independent experiments conducted on HSPC samples obtained from three healthy individual doners.

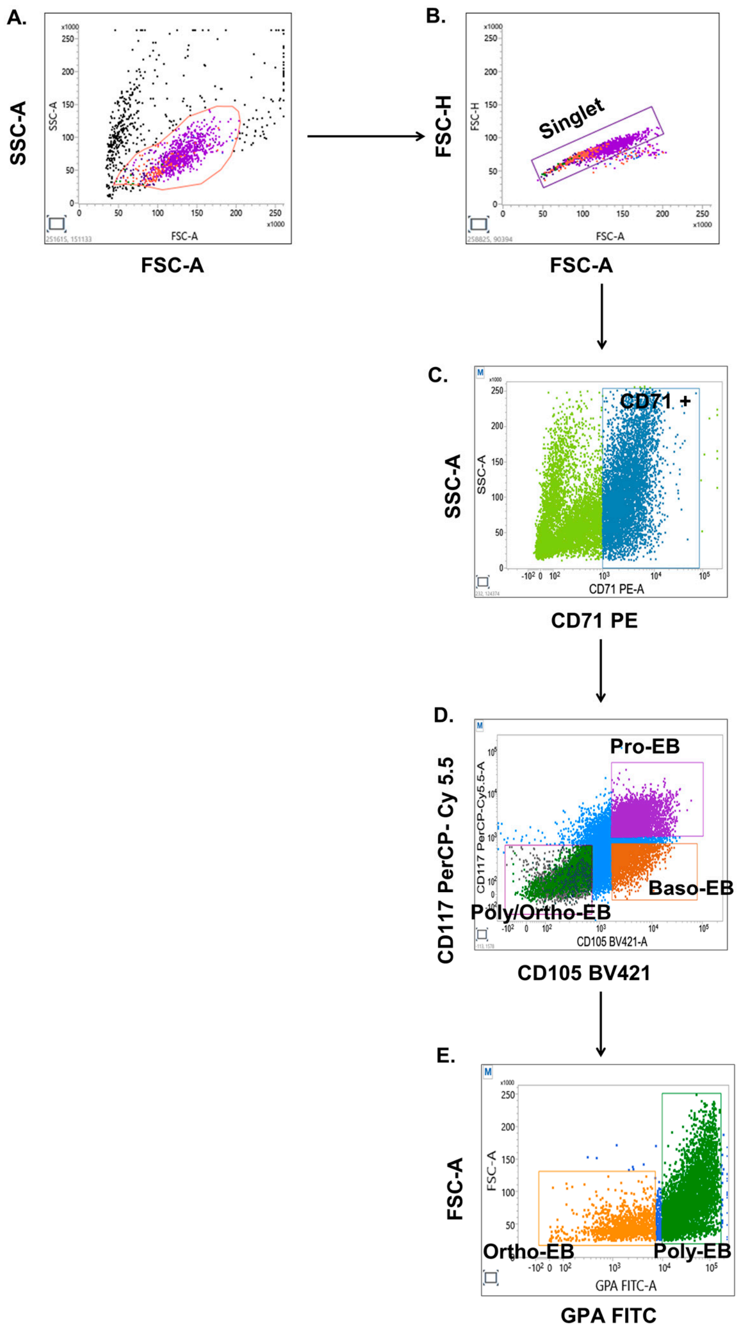

Figure S5: Erythroid panel gating strategy

Representative FACS plots are shown from the analysis of cells harvested on day 18 of erythroid culture. Harvested cells were stained for markers CD71, CD117, CD105 and GPA. The gating strategy identifies cells in (A) forward/side scatter plot (FCS-A and SSC-A) and then single cells are discriminated from doublets using FSC-A/FSC-H plots (B). (C) CD71+ve cells are gated to identify (D) CD105+CD117+ pro-erythroblasts (Pro-EB) , CD105+CD117- basophilic erythroblasts (Baso-EB) and Polychromatophilic erythroblasts / Orthochromatophilic erythroblasts (Poly/Ortho-EB) in the CD105-CD117- region .CD105-CD117- cells were further sub-gated to identify (E). GPA-high FSC high cells as Poly-EB while GPA dim /intermediate and FSC low cells are identified as Ortho-EB.

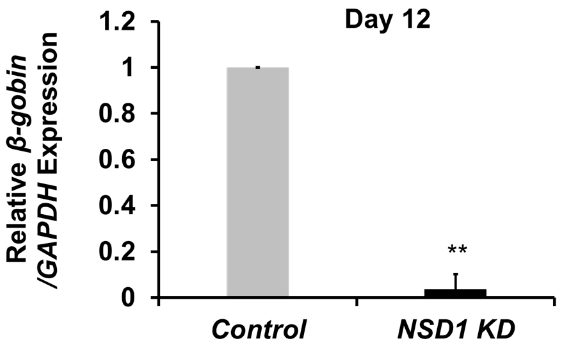

Figure S6: KD of *NSD1* affects *β-globin* gene expression. Relative *β-globin* mRNA expression with *GAPDH* as an internal control upon KD of *NSD1* at day 12 of differentiation. Error bars indicate the SEM of three independent experiments. Error bars indicate mean ± SD of (n=3) independent experiments conducted on HSPC samples obtained from three healthy individual doners. *P<0.05, **P<0.005, ***P<0.0005.

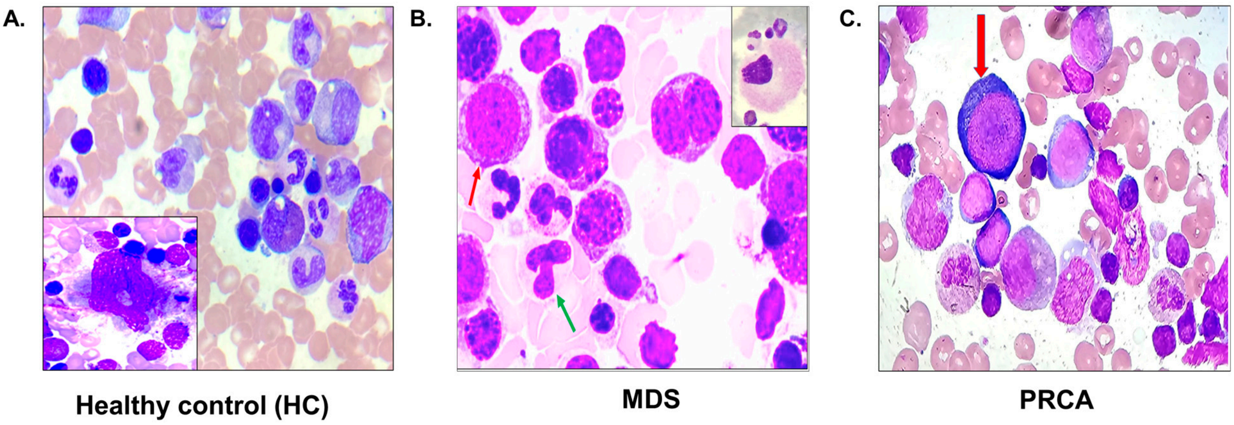

Figure S7: MGG morphology of bone marrow smears of HC v/s MDS/PRCA patients: (A) MGG stained bone marrow aspirate smears show normal trilineage hematopoiesis in a healthy marrow, Inset- Megakaryocyte. (B) Bone marrow examination from a case of myelodysplastic syndrome (MDS) displays megaloblastic erythroid cells (Red arrow), some showing nuclear budding and binucleation; dyspoietic myeloid series cell (green arrow), Inset- Dyspoietic monolobated Megakaryocyte. (C) Bone marrow examination of PRCA showing marked paucity of mature erythroid series cells with presence of only few giant pronormoblast (Red arrow). Images indicative of 100x magnification.

Table S1: List of TaqMan Probes and Primers /SYBR Green Primers used in RT-qPCR for figure S1

Target	Sequence
h β-globin	For: 5′AGGGAGGGCTGAGGGTTTGA-3′
	Rev: 5′CAGGGTGAGGTCTAAGTGATGACA-3′
	Probe: FAM/TCCAACTCCTAAGCCAG-TGCCAGAAGAGCC/BHQ1
h ε-globin	For: 5′AGAGAGGCAGCAGCACATATCTG-3′
	Rev: 5′CATCTTGCTCCACAGGCTAGTGA-3′
	Probe: FAM/AGCTGCAATCACTAGCAA-GCTCTCAGGCC/BHQ1
h γ-globin	For: 5′GGCTGGCTAGGGATGAAGAATAAA-3′
	Rev: 5′TGGCGTCTGGACTAGGAGCTTA-3′
	Probe: FAM/CCTTCAGCAGTTCCACACAC-TCGCTTCTGG/BHQ1
h GAPDH	For: 5′GTGGTCTCCCTGACTTTCAACAGC-3′
	Rev: 5′A TGAGGTCCACCTGCTTGCTG-3′
h GATA1	For: 5′ACCTGCACTGCCTTCATCACT-3′
	For: 5′ GAATACCCCCGGGACTGAAA-3′
h KLF1	For: 5′ GGTTGCGGCAAGAGCTACA-3′
	For: 5′ TGTGTCCCCTCTTCGGTATGC-3′
h GATA2	For: 5′ GCCTCTACTACAAGCTGCACAATG-3′
	For: 5′ TCCTTCCCTAGGTCTGAGCC-3′
h Myogenin	For: 5′GGAACCCCGCTTCTATGATG-3′
	Rev: 5′AAGCTTGGTGGTCCGATGC-3'

Table S2: List of other antibodies used

Antibody	Source	Catalogue Number
Super Bright 600 anti-Human CD133	eBioscience™	63-1338-41
FITC anti-Human CD41a	BD Pharmingen™	555466
Alexa Fluor® 488 anti-Human Ki-67	BD Pharmingen™	561165
PE/Cyanine7 anti-Human CD31	eBioscience™	303117
